# Beyond adherence: refractory dyslipidaemia, the mediterranean diet, and the case for personalized diet-drug complementarity

**DOI:** 10.3389/fnut.2026.1875074

**Published:** 2026-06-22

**Authors:** Roko Skrabic, Marko Kumric, Roko Santic, Ivna Skrabic, Nikola Pavlovic, Doris Rusic, Josko Bozic

**Affiliations:** 1 Department of Internal Medicine, Division of Nephrology and Dialysis, University Hospital of Split, Split, Croatia; 2 Department of Pathophysiology, University of Split School of Medicine, Split, Croatia; 3 Laboratory for Cardiometabolic Research, University of Split School of Medicine, Split, Croatia; 4 Department of Paediatrics, University Hospital of Split, Split, Croatia; 5 Department of Pharmacy, University of Split School of Medicine, Split, Croatia

**Keywords:** dyslipidaemia, gut microbiota, LDL cholesterol, lipoprotein(a), mediterranean diet, non-responder, PCSK9 inhibitors, statins

## Abstract

Cardiovascular disease (CVD) remains the leading cause of death worldwide, and atherogenic dyslipidaemia is one of its most modifiable drivers. The Mediterranean diet (MedDiet) is among the most extensively studied and best-supported dietary patterns for cardiovascular protection, yet meta-analyses show only modest and heterogeneous effects on the conventional lipid panel: typically 5%−15% reductions in LDL cholesterol, 10%−20% reductions in triglycerides and 3%−6% increases in HDL cholesterol, with very high between-study heterogeneity (*I*^2^ often >90%). Lipoprotein(a) [Lp(a)] is genetically determined and largely diet-resistant. Rather than re-asking whether the MedDiet “works”, this narrative review addresses a clinically more useful question: who are the lipid non-responders to a well-adhered MedDiet, and how should the diet be combined with contemporary lipid-lowering pharmacotherapy to close the residual-risk gap? Drawing on PubMed, Scopus and Web of Science (January 2015–April 2026) and seminal earlier trials, we synthesize five themes: genetic and metabolic non-responder phenotypes; the gut microbiota–metabolome axis; inflammaging; MedDiet on top of statin and non-statin therapy; and the obligatory partnership of physical activity, weight loss and smoking cessation. The MedDiet should be reframed not as an alternative to lipid-lowering drugs but as a foundational lifestyle component, personalized by genotype, inflammatory status, microbiota composition and treatment goals.

## 1 Introduction

The Mediterranean diet (MedDiet) is characterized by extra-virgin olive oil as the main fat source; high intake of fruits, vegetables, legumes and whole grains; moderate fish and fermented dairy intake; and low red and processed meat intake (1, 2). Some traditional MedDiet scores include moderate wine consumption with meals; however, alcohol should not be recommended as a cardiovascular-prevention strategy, and the beneficial components of the MedDiet can be achieved without alcohol. Its association with reduced cardiovascular disease (CVD) risk, first systematically described by Ancel Keys' Seven Countries Study, has been reinforced by half a century of observational and trial evidence (1, 3). CVD remains the leading global cause of mortality, accounting for an estimated 19.8 million deaths in 2022, approximately 32% of all global deaths (4), and atherogenic dyslipidaemia - elevated apolipoprotein-B (apoB)-containing lipoproteins, low high-density lipoprotein cholesterol HDL-C functionality and elevated lipoprotein(a) (Lp(a)) - is one of its most clinically actionable components (5).

Pharmacological lipid lowering, especially with statins, ezetimibe, proprotein convertase subtilisin/kexin type 9 (PCSK9)-targeting agents, bempedoic acid and icosapent ethyl, has transformed the management of dyslipidaemia: each 1 mmol/L reduction in low-density lipoprotein cholesterol (LDL-C) achieved with proven LDL-lowering strategies translates into roughly a 22% relative reduction in major vascular events (6–8). Emerging Lp(a)-directed RNA therapeutics may further change residual-risk management, but cardiovascular-outcome data are still awaited. Pharmacological success has not eliminated residual cardiovascular risk: even patients with low on-treatment LDL-C continue to experience events, partly because inflammation, triglyceride-rich remnants, thrombosis, obesity, diabetes, smoking and Lp(a) are not fully captured by LDL-C alone (9). The Mediterranean dietary pattern - through its effects on apoB-containing lipoproteins, endothelial function, oxidative stress, low-grade inflammation, gut microbiota composition and postprandial metabolism - is therefore best viewed as a lifestyle component that complements rather than competes with lipid-lowering drugs (10–13).

Several recent systematic reviews and meta-analyses have already established the average magnitude of MedDiet effects on the lipid panel. Bruna-Mejias et al. pooled 12 metabolic-syndrome trials and reported lower triglycerides (mean difference [MD] −22.4 mg/dL, 95% confidence interval [CI] −32.9 – −11.9), waist circumference and Homeostatic Model Assessment for Insulin Resistance (HOMA-IR), but with very low Grading of Recommendations Assessment, Development and Evaluation (GRADE) certainty and high heterogeneity (*I*^2^ statistic 91%−99%) (14). Hareer et al.'s umbrella review of 18 meta-analyses across 238 randomized controlled trials (RCTs) found that the MedDiet was associated with a 10%−67% reduction in fatal CVD and 21%−70% reduction in non-fatal CVD, but with low or very low certainty and wide effect ranges driven by adherence, comparator and population characteristics (15). This does not imply superiority over all other healthy dietary patterns, such as DASH, Portfolio or well-planned plant-based diets; rather, the MedDiet is used here as a clinically established model for studying diet–drug complementarity in dyslipidaemia. Importantly, none of these syntheses adequately addresses the clinically decisive questions: which patients fail to respond, why they fail, and how MedDiet outcomes change once contemporary lipid-lowering drugs are layered on top. The present review is built around these questions.

Three contextual observations frame the analysis. First, lifestyle changes never act in isolation: in the INTERHEART case-control study of myocardial infarction across 52 countries, nine modifiable factors - including unhealthy diet, physical inactivity, abdominal obesity, smoking and psychosocial stress - together explained more than 90% of the population-attributable risk for first myocardial infarction (16). Diet alone, however nutrient-dense, cannot offset concurrent smoking, sedentariness or abdominal obesity. Second, individual response to a Mediterranean dietary pattern is highly heterogeneous and is shaped by polymorphisms in APOE, PCSK9, PNPLA3, CETP and the LPA gene, by insulin resistance and chronic inflammation, and by the composition of the gut microbiota and its metabolites (17–19). Third, residual-risk phenotypes such as elevated Lp(a) require a framework in which dietary optimisation is paired with risk-appropriate pharmacological prevention (20, 21).

The aims of this review are to define the lipid effects of the MedDiet; identify genetic, metabolic, inflammatory and microbial determinants of non-response; summarize evidence in patients receiving lipid-lowering therapy; and integrate physical activity, weight loss and smoking cessation. We also propose a personalized diet–drug framework consistent with European Society of Cardiology/European Atherosclerosis Society (ESC/EAS) and American College of Cardiology/American Heart Association (ACC/AHA) guidance (22–24). In doing so, we aim to move past the now well-rehearsed question of whether the MedDiet “lowers cholesterol” and toward the clinically useful question of how, and in whom, it should be deployed alongside modern lipid-lowering therapy.

## 2 Methods

This article is presented as a SANRA-informed critical narrative review rather than a systematic review or meta-analysis (25). Because the research question spans mechanistic biology, clinical efficacy, pharmacological complementarity and omics-level mediators, the review was designed to provide a structured critical synthesis rather than a single homogeneous quantitative estimate. The synthesis was organized around five themes: (i) realistic lipid effects of the Mediterranean diet; (ii) genetic, metabolic, inflammatory and microbial determinants of lipid non-response; (iii) lipoprotein (a) and other residual-risk phenotypes; (iv) complementarity between the Mediterranean diet and contemporary lipid-lowering pharmacotherapy; and (v) the role of physical activity, weight loss and smoking cessation as obligatory co-interventions.

PubMed/MEDLINE, Scopus and Web of Science were searched between 1 January 2015 and 25 April 2026, using combinations of Medical Subject Headings (MeSH) and free-text terms: “Mediterranean diet”, “olive oil”, “polyphenols”, “omega-3”, “adherence”, combined with “lipid profile”, “LDL cholesterol”, “HDL cholesterol”, “triglycerides”, “lipoprotein(a)”, “apolipoprotein B”, “statin”, “PCSK9”, “ezetimibe”, “bempedoic acid”, “icosapent ethyl”, “inclisiran”, “pelacarsen”, “olpasiran”, “gut microbiota”, “TMAO”, “short-chain fatty acids”, “inflammaging”, “APOE”, “PNPLA3” and “CETP”. Reference lists of retrieved articles, recent meta-analyses (14, 15, 26), the 2019 ESC/EAS dyslipidaemia guideline, its 2025 focused update and the 2026 ACC/AHA multisociety dyslipidaemia guideline (22–24) were screened manually.

Eligible evidence included randomized controlled trials, prospective cohort studies, large cross-sectional analyses relevant to lipid or lipoprotein phenotypes, Mendelian-randomisation studies, systematic reviews, meta-analyses, guideline documents and expert consensus statements. Mechanistic *in-vitro* and animal studies were used only to support pathophysiological interpretation and were not treated as clinical evidence of efficacy. Seminal trials published before 2015, including the Lyon Diet Heart Study, the original PREDIMED trial and the INTERHEART case-control study, were retained because their findings on dietary patterns and modifiable cardiovascular risk remain foundational and are cited in subsequent guidelines (16, 27–29). Studies were excluded if the dietary intervention did not approximate a Mediterranean dietary pattern, if lipid, lipoprotein, inflammatory, microbiome or pharmacotherapy-relevant outcomes were not reported, if the full text could not be retrieved, or if the full text was not available in English.

Titles, abstracts and full texts were screened independently by R.Sk. and M.K. The final set of included studies, key guideline documents and borderline inclusions were verified by J.B. Disagreements about eligibility or interpretation were resolved by consensus among R.Sk., M.K. and J.B. For included studies, data were extracted narratively and checked by at least two authors. Extracted items included study design, population, intervention and comparator, intervention duration, adherence assessment, background lipid-lowering therapy, lipid or lipoprotein endpoints, main effect estimates and limitations relevant to interpretation. Evidence was then grouped thematically by lipid endpoint, non-responder phenotype and diet-drug complementarity rather than pooled statistically.

Because this was a SANRA-informed narrative review and not a systematic review, no formal study-level risk-of-bias tool, GRADE assessment or meta-analysis was applied. Instead, methodological limitations were considered qualitatively during interpretation, with greater weight given to randomized controlled trials with clinical outcomes, large prospective cohorts, Mendelian-randomisation analyses where causal inference was relevant, high-quality systematic reviews and contemporary guideline documents. When included systematic reviews or meta-analyses reported heterogeneity, risk-of-bias, A MeaSurement Tool to Assess systematic Reviews 2 (AMSTAR-2) or GRADE findings, these were retained and discussed in the synthesis.

The narrative design allowed integration of heterogeneous mechanistic, clinical and pharmacological evidence, but it also introduces potential selection and interpretation bias. The search strategy was broad but was not designed to fulfill all PRISMA requirements for systematic reviews, and the absence of formal pooled estimates limits direct comparison of effect sizes across dietary and pharmacological interventions. Exclusion of non-English full-text articles may have introduced language bias. Cross-study comparability is further limited by differences in Mediterranean diet definitions, adherence instruments, comparator diets, baseline diet quality, extra-virgin olive-oil or nut dose, background medication use, baseline lipid phenotype, geographic setting and intervention duration.

### 2.1 Pathophysiological mechanisms and the limits of dietary modulation

Atherogenic dyslipidaemia is the integrated phenotype of elevated apoB-containing lipoproteins (LDL, very-low-density lipoprotein and remnants), low and dysfunctional HDL-C, elevated triglyceride-rich lipoproteins, and elevated Lp(a) (5, 30). The MedDiet acts on this phenotype through several converging mechanisms which are summarized below; the emphasis here is on the points at which dietary effects saturate or fail, because these are the points at which pharmacological support becomes mandatory.

### 2.2 LDL cholesterol

**Figure 1:**
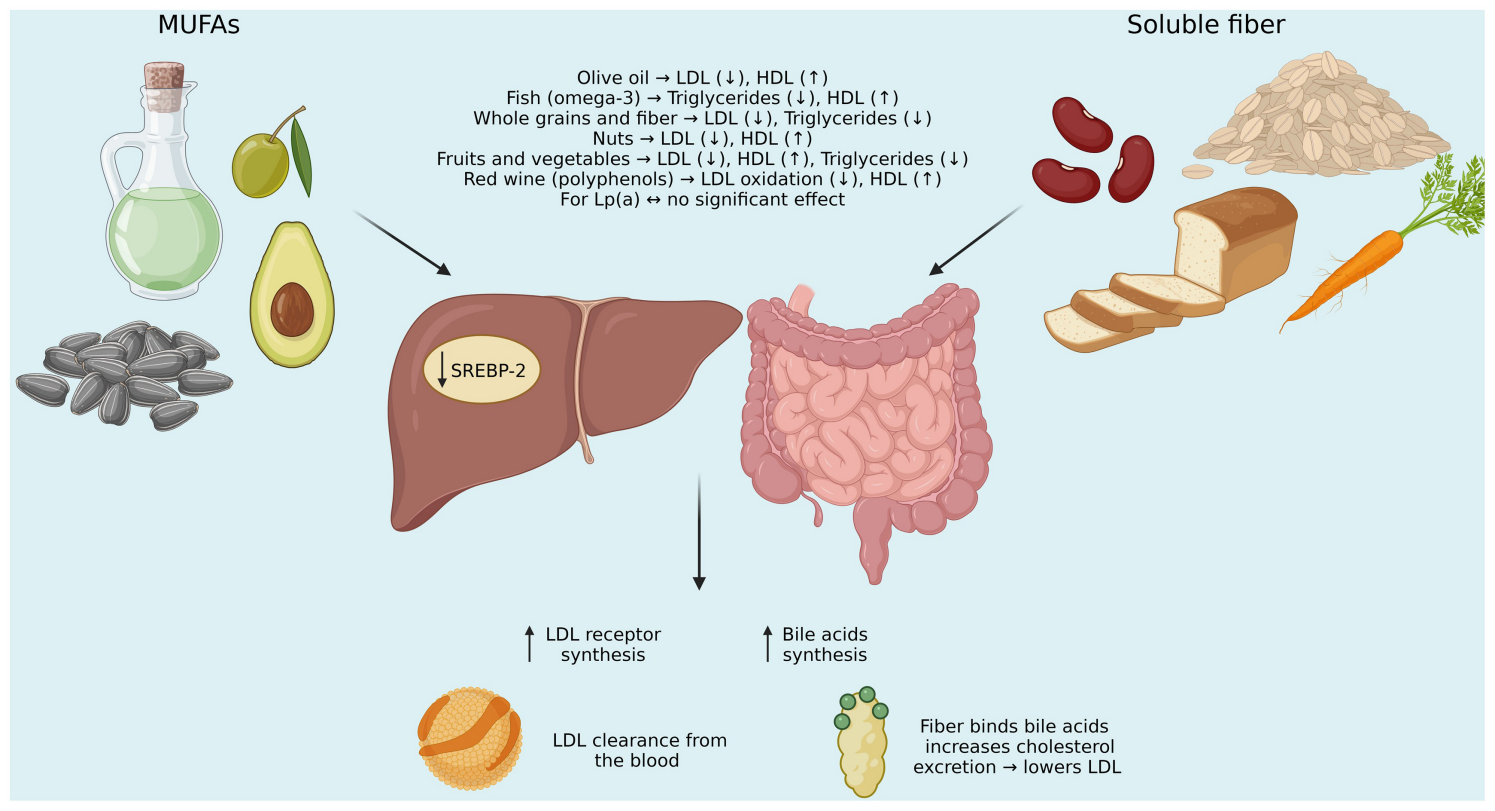
Dietary Modulation of Lipid Metabolism: Mechanisms of the Mediterranean Diet. This figure visually illustrates how components of the Mediterranean diet, specifically monounsaturated fatty acids (MUFAs) and soluble fiber, impact lipid metabolism at both the molecular and physio-logical levels. MUFAs from foods like olive oil, avocado, and nuts downregulate SREBP-2 in the liver, increasing LDL receptor expression and promoting LDL clearance from the bloodstream. Mean-while soluble fiber from sources such as oats, legumes, and vegetables binds bile acids in the gut, enhancing their excretion and prompting hepatic conversion of cholesterol into new bile acids, thus lowering plasma LDL levels. Additional annotations explain the effects of various Mediterranean foods on lipid markers, including reductions in LDL and triglycerides and increases in HDL, while noting the limited impact on lipoprotein(a) [Lp(a)]. Created in https://BioRender.com.

Plasma low-density lipoprotein cholesterol (LDL-C) reflects the balance between hepatic cholesterol synthesis, mediated by 3-hydroxy-3-methylglutaryl-coenzyme A (HMG-CoA) reductase, and low-density lipoprotein receptor (LDLR)-mediated clearance. LDLR expression is regulated mainly by sterol regulatory element-binding protein 2 (SREBP-2), while LDLR recycling is reduced by proprotein convertase subtilisin/kexin type 9 (PCSK9)-mediated degradation (31, 32). Diets high in saturated fatty acids reduce hepatic LDLR activity and raise LDL-C; replacing saturated fatty acids with cis-monounsaturated fatty acids (MUFAs) - the central lipid swap of the MedDiet - modestly increases LDLR-mediated clearance and lowers LDL-C (33). Quantitatively, however, this dietary lever is small: pooled analyses of isocaloric MUFA-for-saturated-fat substitution predict LDL-C reductions of approximately 0.10–0.20 mmol/L (about 4%−8%) (33, 34). Soluble fiber and plant sterols can contribute additional, more modest reductions in LDL-C through bile-acid sequestration and reduced intestinal cholesterol absorption, respectively (35). Statin monotherapy at moderate intensity, by contrast, lowers LDL-C by 30%−40% and high-intensity statin therapy by at least 50%, with each 1 mmol/L absolute reduction translating into a roughly 22% reduction in major vascular events (6, 8). These quantitative differences explain why MedDiet alone is unlikely to achieve LDL-C targets in patients with baseline LDL-C above 3.5 mmol/L, familial hypercholesterolaemia or established atherosclerosis (22–24). These complementary hepatic and intestinal pathways are summarized schematically in Figure 1.

Genetic non-response is the second major constraint. Large genome-wide and Mendelian-randomisation analyses have shown that lipid responses to dietary fat modification vary by APOE genotype, familial or polygenic hypercholesterolaemia and other LDLR-pathway variants (17, 36, 37). PCSK9 loss-of-function variants illustrate the causal importance of LDLR recycling for lifelong LDL-C exposure, but they should not be framed as a diet-resistant dyslipidaemia phenotype (37). Within PREDIMED-Plus and related dietary-intervention studies, lipid responses varied substantially, and exploratory responder analyses support caution against a universal “one in four” estimate: Mediterranean dietary counseling in 223 hyperlipidaemic children classified 156 participants (70%) as LDL-C/non-HDL-C responders and 67 (30%) as non-responders, while a controlled isoenergetic MedDiet study in 26 men with metabolic syndrome identified 11 HDL-C responders (42%) and 8 HDL-C non-responders (31%) (38–40). Mechanistically aligned dietary-fat intervention studies show similarly wide variability RISSCI-1 reported individual LDL-C changes from −1.39 to +0.77 mmol/L after replacing saturated with unsaturated fat, and O'Connor et al. divided 104 participants into LDL-C responder and non-responder tertiles indicating that dietary lipid non-response is endpoint-, threshold- and phenotype-dependent rather than a fixed prevalence estimate (41, 42).

### 2.3 Triglycerides and triglyceride-rich lipoproteins

Plasma triglyceride concentrations reflect hepatic *de-novo* lipogenesis and intestinal chylomicron secretion, both of which are amplified by insulin resistance, refined-carbohydrate intake and excess alcohol (43). The MedDiet's low glycaemic load, high fiber intake and replacement of refined starches by legumes and whole grains lower fasting and post-prandial triglycerides most consistently in patients with insulin resistance or hypertriglyceridaemia (14, 26). Long-chain n-3 polyunsaturated fatty acids (PUFAs), particularly eicosapentaenoic acid (EPA) and docosahexaenoic acid (DHA), may modestly lower triglycerides; however, predictable 20%−30% reductions generally require prescription omega-3 fatty acids at pharmacological doses, as tested with icosapent ethyl in REDUCE-IT (44, 45). Patients with severe hypertriglyceridaemia or familial chylomicronaemia syndrome may require fibrates, prescription omega-3 fatty acids or, in selected severe genetic phenotypes, APOC3-directed therapies (46). PNPLA3 variants, by contrast, mainly modify hepatic-fat susceptibility and may influence metabolic response to dietary intervention (47).

### 2.4 HDL cholesterol—quantity vs. function

HDL particles mediate reverse cholesterol transport and exert pleiotropic vasoprotective effects; quantity (HDL-C) and quality (cholesterol efflux capacity, anti-oxidant and anti-inflammatory function) are imperfectly correlated (48). The MedDiet increases HDL-C only modestly but may improve HDL functionality, including macrophage-specific cholesterol efflux capacity and HDL oxidation status, particularly in high-cardiovascular-risk individuals (49). These effects should be attributed to the dietary pattern as a whole rather than to alcohol or red-wine resveratrol alone; alcohol should not be recommended for cardiovascular prevention. The mixed and often disappointing results of HDL-C-raising strategies, including several CETP-inhibitor programmes, reinforce the point that HDL-C concentration alone is a weak therapeutic target, whereas HDL function remains a plausible mechanistic pathway (48, 50).

### 2.5 Lipoprotein(a): a diet-resistant residual-risk factor

Lp(a) is an LDL-like particle in which apolipoprotein(a) is covalently linked to apolipoprotein B-100; circulating concentrations are 80%−90% heritable, dictated primarily by kringle IV type 2 copy-number variation in the LPA gene (20). Mendelian-randomisation evidence has firmly established Lp(a) as a causal risk factor for atherosclerotic CVD, calcific aortic-valve disease and ischaemic stroke (20, 21). Crucially, Lp(a) is largely refractory to lifestyle interventions: MedDiet adherence, weight loss and exercise are not expected to lower Lp(a) mass concentration, and statins have been shown to modestly raise Lp(a) in pooled analyses (20, 51). In the INTERASPIRE study of 4,367 patients with coronary heart disease, Lp(a) levels showed wide global variation; this study should not be cited as direct evidence that dietary pattern has no effect on Lp(a) unless such an analysis is explicitly reported (52). PCSK9 monoclonal antibodies lower Lp(a) by approximately 20%−30% (53); pelacarsen and olpasiran achieve large Lp(a) reductions in clinical trials, but cardiovascular-outcome data from Lp(a)HORIZON and OCEAN(a)-Outcomes are required before these agents can be integrated into routine practice (51, 54, 55). MedDiet's role in patients with elevated Lp(a) is therefore indirect: improvement of global cardiometabolic, inflammatory and lifestyle-related risk while Lp(a)-specific pharmacological strategies are evaluated.

### 2.6 Non-responder phenotypes: who fails to benefit, and why

Across MedDiet trials, a substantial subgroup of participants has limited lipid improvement despite apparently good adherence (14, 26, 28). For the purpose of this review, lipid non-response refers to failure to achieve a clinically meaningful improvement in the target lipid abnormality after documented high adherence to a Mediterranean dietary pattern for at least 12–24 weeks. This should be distinguished from failure to reach guideline LDL-C targets, which may occur despite an appropriate dietary response when baseline apoB/LDL-C risk is high.

Working definition: a MedDiet lipid non-responder is a patient who, despite documented high adherence for 12–24 weeks, shows minimal improvement in the primary lipid abnormality (for example, < 5% LDL-C reduction, < 10% triglyceride reduction, or persistent above-target apoB/non-HDL-C). This label should be used cautiously because failure to reach a guideline target is not identical to absence of dietary response.

### 2.7 Genetic and Lp(a)-driven non-response

Common variants in APOE (especially the epsilon4 allele), PNPLA3 (rs738409 G), CETP and the FADS1/2 cluster, together with familial or polygenic hypercholesterolaemia, explain part of the inter-individual variability in LDL-C, triglyceride and HDL-C responses to dietary intervention (17–19, 36). Patients with monogenic familial hypercholesterolaemia represent the extreme end of this spectrum: rigorous MedDiet adherence may improve overall dietary quality but is rarely sufficient to achieve LDL-C or apoB targets without high-intensity statin therapy, ezetimibe and, when indicated, PCSK9-targeted therapy (36, 56).

Patients with elevated Lp(a) should not be considered MedDiet non-responders in the conventional lipid-panel sense; rather, Lp(a) represents a genetically driven residual-risk marker requiring aggressive control of LDL-C/apoB and other modifiable risk factors while Lp(a)-directed outcome trials mature (54, 55, 57).

### 2.8 Metabolic and inflammatory non-response

In adults with type 2 diabetes, metabolic syndrome or non-alcoholic fatty liver disease, hepatic *de-novo* lipogenesis and intestinal chylomicron overproduction sustain hypertriglyceridaemia despite high MedDiet adherence (43, 58). In the one-year PREDIMED-Plus results, an energy-restricted MedDiet combined with physical activity and behavioral support improved triglycerides, HDL-C, glycated hemoglobin (HbA1c) and body-weight-related risk factors more than advice to follow an unrestricted MedDiet (38). Weight loss, reduced refined-carbohydrate intake and improved insulin sensitivity are therefore major drivers of triglyceride improvement in this phenotype.

Chronic low-grade inflammation - quantifiable as elevated high-sensitivity C-reactive protein (hs-CRP), interleukin-6 (IL-6) or tumor necrosis factor-alpha (TNF-alpha) - independently predicts cardiovascular events even in patients with well-controlled LDL-C (59). MedDiet adherence has been associated with reductions in inflammatory biomarkers, but effect sizes vary substantially across populations, baseline inflammation, weight change and co-interventions (60). A subgroup of patients with autoimmune disease, chronic kidney disease or persistent obesity retain residual inflammation despite lifestyle optimisation. In selected patients with established atherosclerotic cardiovascular disease (ASCVD), anti-inflammatory strategies such as colchicine 0.5 mg daily have reduced events in outcome trials, but they should be discussed as residual-inflammatory-risk therapy rather than as lipid-lowering treatment (61, 62).

### 2.9 Behavioral non-adherers

Apparent MedDiet non-response may also reflect incomplete or unsustained adherence. Long-term adherence is shaped by socioeconomic status, food availability, ingredient cost, cultural fit and food-environment factors (63, 64). Digital adherence-support tools and structured counseling may help in selected settings, but implementation strategies should be tested in the relevant population and health-care context; in clinical practice, non-response should be interpreted only after adherence, weight change, medication background and secondary causes of dyslipidaemia have been reviewed (65).

### 2.10 The Mediterranean diet in patients receiving lipid-lowering therapy

The decision to initiate statin therapy does not - and should not - end the dietary conversation. Available evidence supports a complementary role for the MedDiet on top of lipid-lowering therapy, but direct trials testing diet-drug interaction with contemporary non-statin regimens remain limited.

In the original PREDIMED trial, many participants were already receiving cardiometabolic medications, including statins, and the olive-oil- or nut-supplemented MedDiet reduced major cardiovascular events relative to a low-fat control diet (27). Because PREDIMED was not designed primarily as a statin-interaction trial, this finding should be interpreted as support for added lifestyle benefit in a medically treated high-risk population rather than definitive proof of statin-specific complementarity. CORDIOPREV provides stronger secondary-prevention evidence that a MedDiet can improve clinical outcomes and vascular/endothelial markers compared with a low-fat diet in patients with established coronary heart disease receiving contemporary medical therapy (66, 67).

The REPRIEVE trial offered an instructive parallel: in 7,769 people living with HIV at low-to-moderate CVD risk, daily pitavastatin reduced major adverse cardiovascular events by 35% over 5.1 years, in a population with a strong residual-inflammation phenotype (68). REPRIEVE did not test the MedDiet directly. Its relevance here is more limited but useful: LDL-C lowering reduces events even in inflammatory phenotypes, while residual inflammatory risk may persist and may require additional lifestyle, metabolic or pharmacological approaches (9, 60, 69).

Whether the MedDiet improves statin-associated muscle symptoms (SAMS) remains uncertain: observational Moli-sani data support diet–statin complementarity for mortality and low-grade inflammation but did not assess SAMS, while SAMS-focused trials of vitamin D and coenzyme Q10 show mixed or negative effects, and blinded n-of-1 data demonstrate a substantial nocebo component (70–75). Therefore, although the MedDiet remains appropriate for cardiometabolic risk reduction in statin-treated patients, current evidence is insufficient to recommend it specifically for SAMS prevention or treatment. For genuinely statin-intolerant patients, bempedoic acid - tested in 13,970 statin-intolerant adults in CLEAR Outcomes - reduced major adverse cardiovascular events by 13% over 3.4 years and is now a risk-based non-statin option that can be paired with MedDiet adherence (76).

The integrated message is that a Mediterranean dietary pattern in a statin-treated patient should be regarded not as redundant but as one component of residual-risk reduction after maximally tolerated, guideline-directed lipid lowering has been achieved.

### 2.11 Gut microbiota, metabolome and inflammaging as mediators of diet effects

A growing body of evidence places the gut microbiota and its metabolites between dietary intake and cardiovascular phenotype, providing one of the most plausible explanations for why the cardiovascular benefit of the MedDiet exceeds its quantitative effect on the standard lipid panel (77, 78).

Three metabolic axes are particularly relevant. First, dietary fiber and polyphenols characteristic of the MedDiet are fermented by Bacteroidetes and Firmicutes into short-chain fatty acids (SCFAs)—acetate, propionate, butyrate—which strengthen intestinal barrier integrity, reduce systemic endotoxin exposure, suppress hepatic lipogenesis through adenosine monophosphate-activated protein kinase (AMPK) activation, and improve insulin sensitivity (77, 79, 80). Second, fermentation of resistant starches and bile-acid metabolism modulates the farnesoid X receptor/Takeda G protein-coupled receptor 5 (FXR/TGR5) signaling axis, influencing hepatic LDL-receptor expression and intestinal cholesterol absorption (78). Third, choline, phosphatidylcholine and L-carnitine—abundant in red meat, full-fat dairy and eggs - are converted by gut microbes into trimethylamine, which the liver oxidizes to trimethylamine N-oxide (TMAO). Elevated TMAO is independently associated with incident myocardial infarction, stroke and all-cause mortality across multiple cohorts (81, 82). The MedDiet's low red-meat content typically lowers TMAO-producing taxa, although shorter dietary interventions ( ≤ 6 months) may not consistently reduce circulating TMAO (83).

In the NU-AGE trial, a 12-month MedDiet intervention in 612 older Europeans shifted the gut microbiome toward taxa associated with higher MedDiet adherence, including Faecalibacterium prausnitzii, Eubacterium and Roseburia. It also reduced inflammaging markers, including IL-6, hs-CRP and interferon gamma-induced protein 10 (IP-10), and improved frailty indices (84). More recently, the DIRECT-PLUS trial demonstrated that a polyphenol- and Mankai-enriched “green Mediterranean” diet produced gut-microbiome shifts that statistically mediated improvements in body weight, intrahepatic fat and cardiometabolic biomarkers, including LDL-C and non-HDL-C (85). These data position the gut microbiota as both a mediator of MedDiet effects and a candidate biomarker for predicting individual response—with potential to personalize dietary advice and identify patients who will require pharmacological augmentation.

Inflammaging - the chronic low-grade pro-inflammatory state of older adults - is mechanistically tied to impaired barrier function, dysbiosis and increased lipopolysaccharide translocation (86). The MedDiet may attenuate this axis through SCFA production, potential modulation of TMAO-producing pathways, polyphenol-mediated inhibition of NF-kappaB and changes in anti-inflammatory cytokine signaling (60, 87). The clinical correlate is that the MedDiet appears especially valuable in older patients, although its effects on specific inflammatory and microbial biomarkers are variable and should not be overinterpreted as direct lipid lowering.

### 2.12 Beyond the diet: physical activity, weight loss and smoking cessation

The original title of this work—and the framing of much of the popular literature—implies that “lifestyle” can be reduced to dietary pattern. INTERHEART showed conclusively that this reduction is empirically incorrect: smoking, abdominal obesity, physical inactivity, hypertension, diabetes and psychosocial stress jointly account for the bulk of the modifiable risk for first myocardial infarction across diverse populations (16). The MedDiet does not, on its own, replace any of these levers.

Regular moderate-to-vigorous physical activity (> = 150 min/week) lowers triglycerides, improves insulin sensitivity and reduces visceral adiposity, with effects that are complementary to those of the MedDiet (88). The PREDIMED-Plus protocol - which couples an energy-restricted MedDiet with progressive aerobic and resistance training and behavioral support - illustrates the real-world configuration that delivers measurable cardiometabolic benefit; long-term PREDIMED-Plus analyses support superior weight and body-composition outcomes, while one-year PREDIMED-Plus results also showed improvements in several cardiovascular risk factors (38, 58).

Sustained weight loss of > = 5%−10% in adults with overweight, obesity or metabolic syndrome reduces triglycerides and improves several cardiometabolic risk factors, independent of dietary pattern (89). Smoking cessation produces large cardiovascular-risk reductions through mechanisms that are complementary to lipid lowering and include reduced oxidative modification of LDL particles and improved vascular function (16, 90). These effects are mechanistically distinct from statin therapy and should not be presented as quantitatively interchangeable with pharmacological LDL-C lowering.

The clinical implication is straightforward: counseling about the MedDiet must be embedded within a comprehensive lifestyle intervention that explicitly targets physical activity, body weight, smoking and psychosocial stress, alongside guideline-directed pharmacotherapy. The MedDiet is the dietary component of cardiovascular prevention, not its totality.

### 2.13 Quantitative lipid outcomes in contemporary trials and meta-analyses

Despite the structural and methodological heterogeneity of the underlying literature, several reasonably robust quantitative conclusions can be drawn from contemporary RCTs and meta-analyses, summarized in Table 1.

**Table 1:** Representative contemporary studies of the Mediterranean diet and the lipid profile (2018–2025), categorized by design and population.

Study	Design/N	Population	Lipid outcome (vs. control/baseline)	Key insight	Key trials
Estruch et al., 2018 (PREDIMED)	RCT, *n* = 7,447, median 4.8 y	High-CVD-risk Mediterranean adults	Modest LDL-C and triglyceride reductions; HDL-C increased	Primary-prevention benefit in high-risk adults; not a statin-interaction trial	(27)
Delgado-Lista et al., 2022 (CORDIOPREV)	RCT, *n* = 1,002, 7 y	Established CHD on optimal medical therapy	Improved clinical outcomes and vascular/endothelial markers	MedDiet superior to low-fat diet for secondary prevention even in statin users	(66, 67)
Salas-Salvadó et al., 2019 (PREDIMED-Plus 1-yr)	RCT, *n* = 626, 12 mo	Metabolic syndrome, BMI ≥27	Triglycerides ↓, HDL-C ↑, HbA1c ↓ in IG vs. CG	Energy restriction + activity drive cardiometabolic gains, not the diet alone	(38)
Ghosh et al., 2020 (NU-AGE)	RCT, *n* = 612, 12 mo	Adults aged 65–79 across 5 EU countries	Reduced inflammaging markers; favorable microbiome shift	Supports microbiome-mediated benefit beyond standard lipid changes	(84)
Rinott et al., 2022 (DIRECT-PLUS)	RCT, *n* = 294, 18 mo	Abdominal obesity/dyslipidaemia	LDL-C, non-HDL-C and intrahepatic fat decreased	Microbiome shift mediated cardiometabolic improvements	(86)
Bruna-Mejías et al., 2025	Meta-analysis of 12 RCTs	Metabolic syndrome	Triglycerides −22.4 mg/dL; LDL-C/HDL-C heterogeneous	Very low GRADE certainty; I^2^ 91–99% across lipid outcomes	(14)
Wu et al., 2025	Meta-analysis of RCTs in T2D	Type 2 diabetes adults	HbA1c − 0.31%, FPG − 0.85 mmol/L, BMI − 0.83 kg/m2, LDL-C − 8.06 mg/dL	Supports multifactorial metabolic benefit in type 2 diabetes	(93)
Vamvakis et al., 2025 (HINTreat *post hoc*)	*Post hoc* analysis, *n* = 61, 6 mo	Stage 1 hypertension; lipid-lowering medication excluded	Higher MedDiet score associated with TC − 7.2, TG − 4.1, LDL-C − 6.4 mg/dL	Supportive lifestyle evidence; lipid-lowering drugs were excluded	(94)
Barkas et al., 2025 (INTERASPIRE)	Cross-sectional, *n* = 4,367	CHD patients across 14 countries	Lp(a) showed wide global variation	Contextual residual-risk evidence; not a MedDiet-effect study	(52)
Hareer et al., 2025 (umbrella review)	18 meta-analyses/238 RCTs	Mixed populations	MedDiet → 10–67% ↓ fatal CVD; 21–70% ↓ non-fatal CVD	Broad outcome support, but certainty and review quality were low	(15)

BMI, body mass index; CG, control group; CHD, coronary heart disease; CVD, cardiovascular disease; EU, European Union; FPG, fasting plasma glucose; GRADE, Grading of Recommendations Assessment, Development and Evaluation; HbA1c, glycated hemoglobin; HDL-C, high-density lipoprotein cholesterol; IG, intervention group; I^2^, measure of statistical heterogeneity across studies; LDL-C, low-density lipoprotein cholesterol; Lp(a), lipoprotein(a); MedDiet, Mediterranean diet; mo, months; n, sample size; RCT, randomized controlled trial; TC, total cholesterol; TG, triglycerides; T2D, type 2 diabetes mellitus; vs., versus; y, years.

For LDL-C, pooled RCT estimates converge on average reductions of 5%−15% with strict MedDiet adherence, with the largest effects seen in Mediterranean populations consuming high doses of extra-virgin olive oil or nuts and the smallest in non-Mediterranean and low-adherence settings (14, 15, 26, 27). The 2025 systematic review by Bruna-Mejías et al. of 12 metabolic-syndrome trials reported very heterogeneous LDL-C effects (*I*^2^ = 99%), with some trials showing reductions and others showing neutral or paradoxical findings, again emphasizing the role of patient phenotype rather than diet *per se* (14).

For triglycerides, the MedDiet produces the most consistent reductions in patients with hypertriglyceridaemia, metabolic syndrome or type 2 diabetes and smaller effects in normotriglyceridaemic adults (14, 26, 91, 92). In a 2025 meta-analysis of MedDiet trials in type 2 diabetes, pooled mean differences favoring the MedDiet included reductions in HbA1c (−0.31%), fasting glucose (−0.85 mmol/L), body mass index (BMI) (−0.83 kg/m^2^) and LDL-C (−8.06 mg/dl), consistent with multifactorial cardiometabolic improvement rather than isolated triglyceride lowering (93).

For HDL-C, increases of 3%−6% are typical and clinically modest, but the qualitative improvements in HDL function—efflux capacity, anti-oxidant and anti-inflammatory activity—appear to translate into part of the observed cardiovascular benefit (49, 50).

For Lp(a), no clinically meaningful reduction is expected with the MedDiet, consistent with its strong genetic determinism. PCSK9 monoclonal antibodies lower Lp(a) by approximately 20%−30%, while pelacarsen and olpasiran produce large Lp(a) reductions in clinical trials; however, cardiovascular-outcome data are still required before Lp(a)-directed RNA therapies can be described as outcome-proven treatments (53–55, 57).

A recent *post hoc* analysis by Vamvakis et al. of the HINTreat trial examined adults with stage 1 hypertension after excluding participants who received lipid-lowering medication. In this *n* = 61 analysis, higher MedDiet score was associated with reductions in total cholesterol (*B* = −7.24 mg/dl, *p* < 0.001), triglycerides (*B* = −4.10 mg/dl, *p* = 0.035) and LDL-C (*B* = −6.43 mg/dl, *p* = 0.004) (94). This finding is supportive evidence for diet-associated lipid improvement during intensive lifestyle treatment, but it should not be described as evidence of additive MedDiet effects on top of contemporary lipid-lowering therapy in a secondary-prevention cohort (94).

Adherence remains the rate-limiting variable. In Bonaccio et al.'s Moli-sani analyses, sustained high MedDiet adherence over multiple years was substantially constrained by socioeconomic gradients (63). In non-Mediterranean settings the same dietary pattern is achievable but more demanding behaviorally and economically—a gap that public-health policy, food-environment interventions and digital adherence tools are only beginning to address (64, 65).

## 3 Discussion: a personalized diet-drug framework

**Figure 2:**
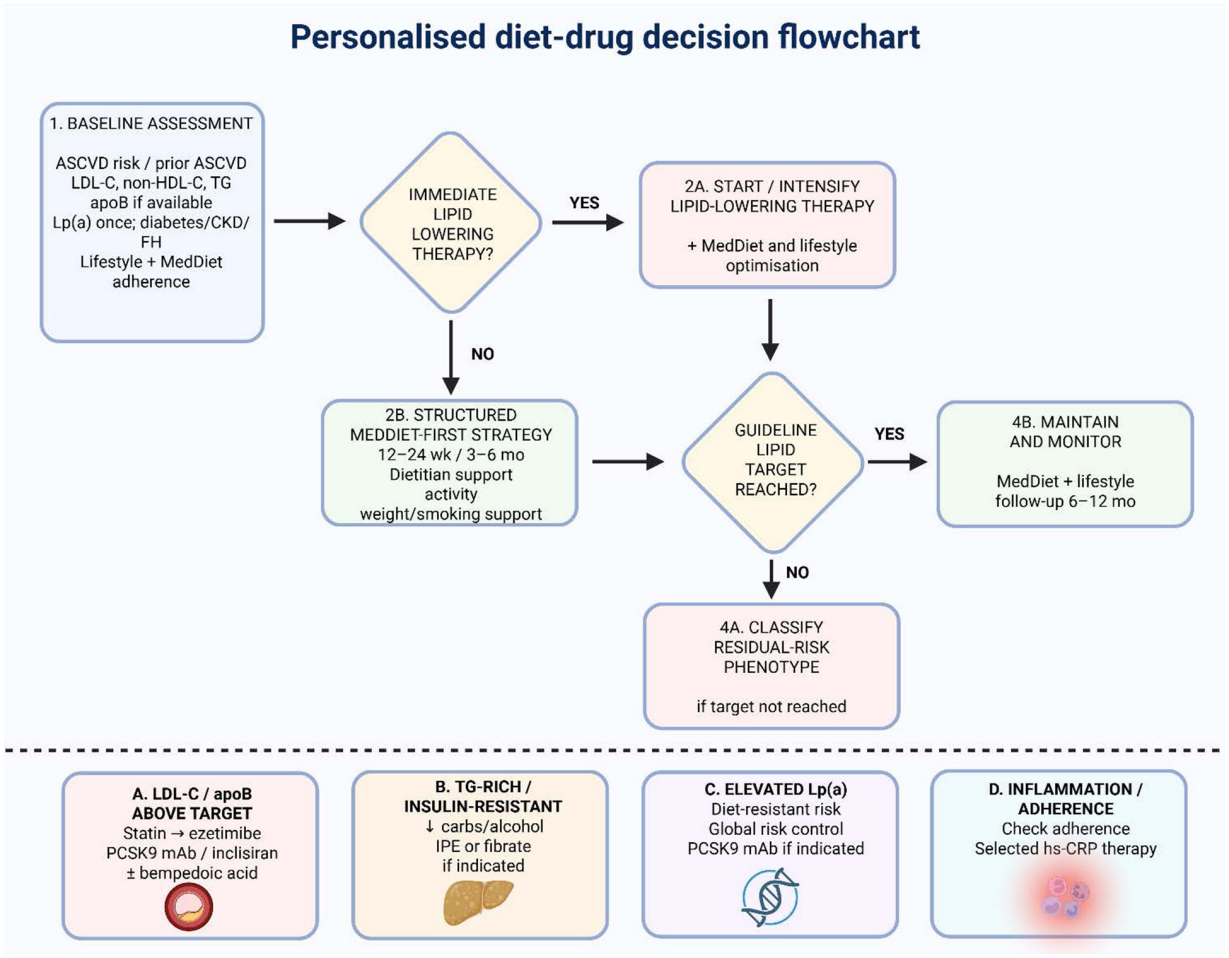
Personalized diet-drug decision flowchart for refractory dyslipidaemia after Mediterranean diet implementation. The figure summarizes a pragmatic clinical framework for integrating the Mediterranean diet (MedDiet) with contemporary lipid-lowering pharmacotherapy in patients with dyslipidaemia. Baseline assessment should include global atherosclerotic cardiovascular disease (ASCVD) risk, previous ASCVD, low-density lipoprotein cholesterol (LDL-C), non-high-density lipoprotein cholesterol (non-HDL-C), triglycerides (TG), apolipoprotein B (apoB) when available, lipoprotein(a) [Lp(a)] at least once in adult life, diabetes, chronic kidney disease (CKD), suspicion of familial hypercholesterolaemia (FH), lifestyle factors and MedDiet adherence. Patients in whom immediate lipid-lowering therapy is indicated—including those with established ASCVD, FH, high-risk diabetes or CKD, severe LDL-C or TG elevation, or major risk-enhancing features—should not undergo a prolonged diet-only trial; lipid-lowering therapy should be started or intensified while MedDiet and lifestyle optimisation continue in parallel. In lower-risk patients without an immediate pharmacological indication, a structured MedDiet-first strategy supported by dietitian counseling, physical activity, weight management and smoking/sedentary-behavior intervention may be reassessed after 12–24 weeks/3–6 months. The second decision point asks whether guideline-based lipid targets have been reached. This refers primarily to LDL-C targets selected according to absolute ASCVD risk and clinical phenotype, with non-HDL-C, apoB and TG considered according to guideline context and the dominant lipid abnormality. If targets are achieved, MedDiet and lifestyle measures should be maintained with periodic follow-up. If targets are not achieved, residual risk should be classified according to the dominant phenotype. Persistent LDL-C/apoB elevation supports stepwise intensification with statin therapy, ezetimibe, PCSK9 monoclonal antibody therapy or inclisiran, with bempedoic acid considered as an add-on or statin-intolerance option. A TG-rich/insulin-resistant phenotype should prompt emphasis on weight loss, reduced refined-carbohydrate and alcohol intake, and phenotype-specific pharmacotherapy, including icosapent ethyl in selected high-risk patients and fibrate therapy when severe hypertriglyceridaemia or pancreatitis risk is present. Elevated Lp(a) is presented as a largely diet-resistant, genetically determined residual-risk modifier requiring aggressive control of all modifiable risk factors; PCSK9 monoclonal antibodies may be considered when otherwise indicated, while dedicated Lp(a)-lowering RNA therapies require outcome-based integration before routine clinical use. The inflammatory/adherence phenotype highlights the need to confirm true MedDiet adherence, address implementation barriers and consider anti-inflammatory risk-reduction strategies in selected ASCVD patients with persistent inflammatory risk, rather than treating inflammation-directed therapy as a lipid-lowering substitute. The residual-risk categories are not mutually exclusive, and more than one mechanism may contribute to persistent above-target risk in an individual patient. Overall, the MedDiet is presented as a foundational lifestyle intervention that complements, rather than replaces, risk-based lipid-lowering pharmacotherapy. Created in https://BioRender.com. apoB, apolipoprotein B; ASCVD, atherosclerotic cardiovascular disease; CKD, chronic kidney disease; FH, familial hypercholesterolaemia; HDL-C, high-density lipoprotein cholesterol; hs-CRP, high-sensitivity C-reactive protein; IPE, icosapent ethyl; LDL-C, low-density lipoprotein cholesterol; Lp(a), lipoprotein(a); MedDiet, Mediterranean diet; non-HDL-C, non-high-density lipoprotein cholesterol; PCSK9, proprotein convertase subtilisin/kexin type 9; TG, triglycerides.

Three integrated insights emerge from the literature reviewed here. First, the average effect of the Mediterranean diet on the standard lipid panel is real but quantitatively limited; pharmacotherapy remains the dominant tool for achieving guideline-recommended LDL-C and non-HDL-C targets in patients with established atherosclerosis, familial hypercholesterolaemia, type 2 diabetes or chronic kidney disease (6, 8, 22–24). Second, the cardiovascular benefit of the MedDiet may exceed what its modest lipid changes alone would predict, because the diet acts concurrently on inflammation, oxidative stress, endothelial function, the gut microbiota and HDL functionality - domains that pharmacotherapy addresses only partially (10, 11, 60, 77, 84). Third, individual response to the MedDiet is highly heterogeneous and is plausibly shaped by genetic, metabolic and inflammatory phenotypes, opening the door to more personalized diet-drug strategies (17, 18, 36). Figure 2 translates this personalized diet–drug framework into a stepwise clinical flowchart, from baseline risk assessment to residual-risk phenotype–guided treatment escalation.

The clinical implication is a shift from a binary “diet vs. drug” framing to a layered intervention model. In low-risk primary-prevention adults, the MedDiet plus physical activity, weight management and smoking cessation may achieve adequate lipid control without pharmacotherapy. In moderate- to high-risk patients, statin intensity and non-statin add-on therapy should be selected according to absolute ASCVD risk, baseline LDL-C/apoB, tolerance and guideline targets, while the MedDiet contributes inflammatory, microbial and functional benefits not achievable through LDL-C lowering alone (22–24, 28). In very-high-risk or statin-intolerant patients, ezetimibe, bempedoic acid, PCSK9 monoclonal antibodies and, when appropriate, inclisiran can help achieve LDL-C targets. Inclisiran should be described as a twice-yearly LDL-C-lowering therapy with cardiovascular-outcome trials ongoing, not as an outcome-proven therapy (6, 56, 76, 95, 96). In patients with elevated Lp(a), aggressive control of all modifiable risk factors remains the practical strategy until Lp(a)-directed therapies demonstrate outcome benefit and become clinically available (51, 54, 55, 57).

Table 2 summarizes the principal pharmacological tools and their complementarity points with the MedDiet.

**Table 2:** Contemporary lipid-lowering pharmacotherapy and its complementarity with the Mediterranean diet.

Drug class/agent	Mechanism	Approx. LDL-C/lipid effect	Complementarity with MedDiet	Key trial/evidence status
Moderate–high-intensity statin (atorvastatin, rosuvastatin)	HMG-CoA reductase inhibition; ↑ LDLR	LDL-C ↓ 30–55%; triglycerides ↓ 10%−25%	MedDiet may add metabolic, inflammatory and dietary-quality benefit; statin-tolerability benefit remains unproven	CTT meta-analyses (6, 8); PREDIMED (27); CORDIOPREV (66)
Ezetimibe (often statin add-on)	NPC1L1 inhibition → ↓ intestinal cholesterol absorption	Adds 18%−22% LDL-C reduction to statin	MedDiet's plant sterols and fiber act on the same pathway non-pharmacologically	IMPROVE-IT, outcome-proven as statin add-on (100); ESC/EAS/ACC guidance (22–24)
Bempedoic acid	ATP-citrate lyase inhibition; liver-specific	LDL-C ↓ 17%−28%; ↓ hs-CRP	Useful in statin-intolerant patients on a MedDiet; minimal myopathy risk	CLEAR Outcomes, outcome-proven in statin-intolerant/high-risk patients (76)
PCSK9 monoclonal antibodies (evolocumab, alirocumab)	Block PCSK9-mediated LDLR degradation	LDL-C ↓ 50%−60%; Lp(a) ↓ 20–30%	MedDiet provides lifestyle, inflammatory and microbial benefits complementary to apoB/LDL-C lowering	FOURIER (7); ODYSSEY OUTCOMES (101); Lp(a) analysis (53)
Inclisiran (siRNA, twice yearly)	Hepatic PCSK9 mRNA degradation	LDL-C ↓~50%; durable	Twice-yearly dosing may reduce treatment burden; MedDiet remains needed for lifestyle and metabolic risk	ORION-10/11 LDL-C lowering proven (95); cardiovascular-outcome data pending
Icosapent ethyl (high-dose EPA)	Reduces VLDL secretion; anti-inflammatory	Triglycerides ↓ 20%−30%; CV events ↓ 25%	Pairs with carbohydrate quality, weight management and alcohol reduction; not simply habitual fish intake	REDUCE-IT, outcome-proven in selected statin-treated patients (45)
Fibrates (fenofibrate)	PPAR-α agonism	Triglycerides ↓ 20%−30%; HDL-C ↑ 5%−15%	MedDiet low-glycaemic load and weight loss are complementary for TG-rich lipoprotein phenotype	TG lowering is proven, but cardiovascular outcome benefit is inconsistent and phenotype-dependent; the strongest signal has been observed in high-TG/low-HDL-C subgroups, while modern outcome trials have not uniformly confirmed event reduction (102, 103); guideline-selected use (22–24)
Pelacarsen (ASO, phase 3)	Hepatic LPA mRNA degradation	Lp(a) ↓ 70%−80%	MedDiet does not lower Lp(a); role is indirect global-risk control	Lp(a)HORIZON ongoing; Lp(a) lowering proven, outcomes pending (57)
Olpasiran (siRNA, phase 3)	Hepatic LPA mRNA degradation	Lp(a) ↓ 95%−98%; durable	MedDiet does not lower Lp(a); role is indirect global-risk control	OCEAN(a)-DOSE/OCEAN(a)-Outcomes; outcomes pending (54)
Colchicine 0.5 mg (anti-inflammatory; not lipid-lowering)	Inhibits NLRP3 inflammasome/neutrophil activation	CV events reduced in selected ASCVD populations; no lipid-lowering effect	May address residual inflammatory risk separately from MedDiet; not a lipid-lowering substitute	COLCOT (61); LoDoCo2 (62)

ASCVD, atherosclerotic cardiovascular disease; ASO, antisense oligonucleotide; ATP, adenosine triphosphate; CTT, Cholesterol Treatment Trialists' Collaboration; CV, cardiovascular; EPA, eicosapentaenoic acid; HMG-CoA, 3-hydroxy-3-methylglutaryl-coenzyme A; hs-CRP, high-sensitivity C-reactive protein; LDL-C, low-density lipoprotein cholesterol; LDLR, low-density lipoprotein receptor; Lp(a), lipoprotein(a); MedDiet, Mediterranean diet; mRNA, messenger RNA; NLRP3, NOD-like receptor family pyrin domain-containing 3; NPC1L1, Niemann-Pick C1-Like 1; PCSK9, proprotein convertase subtilisin/kexin type 9; PPAR-α, peroxisome proliferator-activated receptor alpha; siRNA, small interfering RNA; TG, triglycerides; VLDL, very-low-density lipoprotein.

Clinical use should be guided by baseline LDL-C, apoB and absolute ASCVD risk, but also by phenotype-specific factors such as familial or polygenic hypercholesterolaemia, fasting triglycerides, diabetes or insulin resistance, hs-CRP and Lp(a). Lp(a) should be measured at least once in adult life, while behavioral adherence should be assessed structurally rather than assumed (22–24).

From a practical precision-nutrition perspective, additional testing should be selective rather than routine: Lp(a) should be measured at least once in adult life, especially in premature ASCVD, family history, suspected familial hypercholesterolaemia or unexplained residual risk, whereas routine LPA genotyping, APOE, PNPLA3, CETP testing, gut-microbiota profiling and TMAO measurement are not currently justified for standard MedDiet personalisation because their clinical utility for directing lipid therapy remains insufficiently validated (18, 20, 22–24). Pharmacotherapy should be prioritized over prolonging dietary intervention in patients with established ASCVD, familial hypercholesterolaemia, diabetes or chronic kidney disease with above-target LDL-C/apoB, markedly elevated baseline LDL-C, elevated Lp(a), or persistent lipid non-response after 12–24 weeks of documented high MedDiet adherence (22–24). Operationally, this means intensifying pharmacotherapy rather than extending diet-only management when LDL-C remains ≥1.4 mmol/L (≥55 mg/dl) in very-high-risk ASCVD, ≥1.8 mmol/L (≥70 mg/dl) in high-risk patients or ASCVD not at very high risk, or ≥2.6 mmol/L (≥100 mg/dl) in borderline/intermediate- or moderate-risk primary prevention; baseline LDL-C ≥4.9 mmol/L (≥190 mg/dl) or Lp(a) ≥125 nmol/L (≥50 mg/dl) should also favor earlier pharmacological intensification (20, 22–24, 36).

MedDiet definitions vary across trials, and adherence is measured with heterogeneous tools, including the Mediterranean Diet Adherence Screener (MEDAS), the PREDIMED 14-item score, the alternate Mediterranean score and the Trichopoulou index, each with different sensitivity to actual intake. The high *I*^2^ values reported in meta-analyses should therefore be interpreted not only as statistical inconsistency but also as clinical and implementation heterogeneity, driven by differences in adherence assessment, extra-virgin olive-oil or nut dose, intervention intensity, energy restriction, physical-activity co-interventions, baseline diet quality of control groups, medication background, metabolic phenotype, geographic setting and intervention duration (14, 15, 26, 27, 38). Most studies focus on surrogate lipid endpoints rather than hard cardiovascular events, and individual responses are rarely reported. Nutrigenomic and metagenomic data are still largely observational and require validation in interventional designs. Finally, generalisability beyond Mediterranean and Western populations remains limited, so future studies should test culturally adapted MedDiet patterns in Asian, African and Latin-American settings.

Future work should move beyond average lipid responses and test whether Mediterranean diet interventions provide incremental cardiovascular benefit when implemented alongside contemporary lipid-lowering regimens, including PCSK9-targeted therapies and emerging Lp(a)-lowering agents. Greater attention is also needed to the clinical utility of polygenic risk scores and microbiome profiling as tools for tailoring dietary advice, rather than as exploratory biomarkers alone. In non-Mediterranean settings, comparative studies should evaluate how the MedDiet performs against other healthy and environmentally sustainable dietary patterns (97, 98). Finally, implementation research should address the practical barriers that determine long-term adherence, particularly food cost, availability and cultural fit, while assessing whether digital-health platforms can provide durable behavioral support beyond the short-term improvements reported in early studies (64, 65).

## 4 Conclusion

The Mediterranean diet remains one of the best-evidenced dietary patterns for cardiovascular prevention, but its effect on the standard lipid panel is modest and highly variable, mediated as much by genetic, metabolic, inflammatory and microbial individuality as by the diet itself. Reframing the diet as a precision-nutrition component (99) - to be combined with risk-appropriate statins, ezetimibe, bempedoic acid, icosapent ethyl, PCSK9 monoclonal antibodies and, where indicated, inclisiran or emerging Lp(a)-directed RNA therapies - reconciles the diet's well-documented epidemiological benefit with its quantitative limits as a stand-alone lipid-lowering intervention. The clinically useful question is no longer whether the Mediterranean diet “works”, but how, and in whom, it should be deployed alongside modern lipid-lowering therapy and comprehensive lifestyle risk reduction. Answering that question for the individual patient is the central task of contemporary cardiometabolic medicine.
